# Clinical impact of different detection methods for disseminated tumor cells in bone marrow of patients undergoing surgical resection of colorectal liver metastases: a prospective follow-up study

**DOI:** 10.1186/1471-2407-10-153

**Published:** 2010-04-20

**Authors:** F Jeroen Vogelaar, Wilma E Mesker, Arjen M Rijken, Gaby W van Pelt, Antonia M van Leeuwen, Hans J Tanke, Rob A Tollenaar, Gerrit J Liefers

**Affiliations:** 1 Department of Surgery, Leiden University Medical Center, Leiden, the Netherlands; 2 Department of Surgery, Amphia Hospital, Breda, the Netherlands; 3 Department of Molecular Cell Biology, Leiden University Medical Center, Leiden, the Netherlands; 4 Department of Pathology, Rijnland Hospital, Leiderdorp, the Netherlands

## Abstract

**Background:**

Large number of patients with colorectal liver metastasis show recurrent disease after curative surgical resection. Identification of these high-risk patients may guide therapeutic strategies. The aim of this study was to evaluate whether the presence of disseminated tumor cells in bone marrow from patients undergoing surgical resection of colorectal liver metastases can predict clinical outcome.

**Methods:**

Sixty patients with colorectal liver metastases were planned for a curative resection between 2001 and 2007. All patients underwent bone marrow aspiration before surgery. Detection of tumor cells was performed using immunocytochemical staining for cytokeratin (CK-ICC) combined with automated microscopy or indirectly using reverse transcription-polymerase chain reaction (RT-PCR).

**Results:**

Disseminated tumor cells were found in 15 of the 46 patients (33%) using CK-ICC and in 9 of 44 of the patients (20%) using RT-PCR. Patients with negative results for RT-PCR had a significant better disease-free survival after resection of their liver metastases (p = 0.02). This group also showed significant better overall survival (p = 0.002). CK-ICC did not predict a worse clinical outcome.

**Conclusions:**

The presence of disseminated tumor cells in bone marrow detected using RT-PCR did predict a worse clinical outcome. The presence of cells detected with CK-ICC did not correlate with poor prognosis.

## Background

Over the past decades surgical resection has evolved as the first choice of treatment for colorectal liver metastases because it is a relatively safe and potentially curative procedure [1,2]. The reported 3-year survival of patients after surgical resection of colorectal liver metastasis ranges from 57% to 73% [3,4]. However, even after curative surgical resection, a high percentage of patients show recurrent disease, either in the liver or extra hepatic, within a relatively short period of time after surgical treatment [5] caused by minimal residual disease (MRD) [6]. These high-risk patients might benefit from additional systemic treatment [7]. Currently available prognostic factors are insufficient to select patients at risk for tumor progression [8]. Therefore the need for additional methods for the selection of high-risk patients is evident.

On a clinical level, the Memorial Sloan-Kettering Cancer Center Clinical Risk Score (MSKCC-CRS) is a frequently used tool to predict the risk for recurrence and tumor progression [4]. On a cellular level, disseminated tumor cells (DTCs) might also give this prognostic information. DTCs can be detected in blood and bone marrow of patients with various epithelial malignancies either directly, using immunocytochemical staining combined with automated microscopy (CK-ICC), or indirectly using reverse transcription-polymerase chain reaction (RT-PCR) [6,9,10]. Automated microscopy is proven to be an accurate method for pathological evaluation of tumor cells in blood and bone marrow [11].

Currently, most data on the prognostic value of DTCs are available for breast cancer. Recent meta-analysis [12] showed that the presence of DTCs in bone marrow was predictive for the development of distant metastases in breast cancer. Tumor cell persistence in bone marrow also showed to be an independent prognostic factor for subsequent breast cancer survival [13].

However, in colorectal cancer the results are more controversial. Different groups describe a positive association between DTCs in bone marrow and an increased recurrence rate or reduced survival while other found no association between DTCs and prognostic factors [6]. Therefore, the clinical meaning of DTC detection in colorectal cancer is still open for debate both for CK-ICC and RT-PCR [14-19].

The aim of this prospective study is to evaluate whether the presence of disseminated tumor cells in bone marrow from patients undergoing surgical resection of colorectal liver metastases is associated with worse overall survival and shorter disease free survival.

## Methods

### Patients

Between October 2001 and November 2007, a total of 180 consecutive patients with colorectal liver metastases were scheduled for surgical therapy. Only the patients planned for curative resection were included in this study. Other types of surgery or diagnoses were excluded: surgery combined with radio frequency ablation (RFA) (n = 25), RFA alone (n = 23), isolated liver perfusion (n = 64), liver perfusion prior to surgery (n = 2), benign disease (n = 2), other malignancy (n = 4). Overall, 60 patients planned for resection of the liver metastases were included for analysis. Twenty four of them were diagnosed in 2001-2004 and 36 were included in 2005-2007. Approval from all the local Ethical Committees for this study was granted and informed written consent was obtained from all patients. All patients underwent a preoperative abdominal computed tomography (CT) to confirm metastatic disease confined to the liver. Eligibility and exclusion criteria for the scheduled treatment and criteria for disease progression within the liver according to the WHO guidelines have been previously published [1,20-23]. During follow-up, CT-scans of the liver were made at 4, 8 and 12 months after surgery and then after every 12 months until 3 years after surgery. The patients who did not undergo any intervention or showed disease progression which could not be surgically treated, were referred to a medical oncologist for further treatment. All patients underwent bone marrow aspiration under general anesthesia just prior to surgery and all patients were followed up until June 2008.

### Bone marrow aspiration

5-10 ml of bone marrow was aspirated from both sides of the anterior iliac crest of all included patients. Before inserting the needle in the anterior iliac crest, an incision was made into the overlying skin to prevent contamination with skin epithelial cells. Mononuclear cells were isolated from bone marrow by ficoll gradient centrifugation and aliquoted to isolate RNA to use for the RT-PCR or to make cytospin-slides to stain with ICC.

### Immunocytochemistry and automated microscopy (CK-ICC)

The cytospin slides were stained with primary antibodies A45-B/B3, directed against cytokeratins 8, 18 and 19 or with isotype control antibodies directed against an irrelevant antigen, MOPC21, as a negative control staining. A detailed protocol has been published before by Pantel et al [24]. This staining resulted in a red precipitate in the cytoplasm of cytokeratin 8, 18 and 19-positive cells. The slides were counterstained with hematoxylin to visualize nuclear morphology. The stained slides were analysed using the ARIOL SL-50 automated microscope^®^. One slide stained for cytokeratin and one negative control slide were analyzed per patient. The features of this system have been previously published [25].

Combining ICC with automated microscopy, cytokeratin-positive cells were confirmed by a independent pathologist and categorized based on morphological criteria according to the guidelines of the European ISHAGE Working Group for Standardization of Tumour Cell Detection [26]. Candidate tumor cells and apoptotic cells were cells that did not meet all criteria for a positive cell but could not be unambiguously defined as normal. A patient was considered positive if at least one tumor cell, candidate tumor cell or apoptotic cell was found, all verified by an independent pathologist.

### Reverse transcription-polymerase chain reaction (RT-PCR)

Total RNA was extracted from the mononuclear cells by Trizol reagent. Random primed cDNA was synthesized from 1 μg of total RNA using the 1^st ^strand cDNA synthesis kit for RT-PCR (AMV). cDNA samples were five times diluted to 100 μl to diminish pipetting variation. Primers and probes for the marker CK20 were selected with Primer Express^®^v1.5 software. The low-copy housekeeping gene porphobilinogen deaminase was used as an internal control. For each patient two RNA samples resulting in cDNA samples were processed. Five micro-liters of cDNA were used per amplification. For all PCRs the same PCR conditions were used. Per reaction 300 nM of each primer was used.

PCR samples were considered positive if the threshold cycle was less than 55. The threshold cycle reflects the PCR cycle number at which the fluorescence generated within a reaction crosses the threshold (background noise). The threshold cycle is inversely proportional to the copy number of the target template i.e. the higher the template concentration, the lower the threshold cycle measured. Bone marrow from a patient was considered positive if at least one of the PCR samples was positive after duplo analysis.

### Statistical analysis

Frequencies were described as mean (SD), or median (range) in case of a non-normal distribution. The mortality of the patients with positive RT-PCR was compared with the subjects with negative RT-PCR using Cox regression adjusted for sex and age. The same analysis was done for CK-ICC. Hazard ratio's (HR) were calculated by Cox regression analysis for disease-related survival and disease-free survival. Disease-related survival was considered from the day of liver metastases-surgery to the day of death due to disease or censored at most recent follow-up visit. Patients who did not undergo resection of the liver metastases, who showed extra-hepatic disease at the time of surgery or who died after the operation due to complications or none disease-related causes were excluded from disease-related survival analyses. Disease-free survival was considered from the day of surgery to the day of recurrence or censored at most recent follow-up. The association between mortality ans ICC or RT-PCR was visually depicted with a Kaplan-Meier survival curve. All analyses were performed with SPSS for Windows (version 16.0, SPSS Inc, Chicago, Ill). P-values of less than 0.05 were considered statistically significant.

## Results

The study population comprised of 42 males and 18 females. No complications of bone marrow aspiration were reported. Overall, 27% of the patients (16/60) did not undergo the planned surgical treatment because of the presence of extra-hepatic disease (n = 9), the high number of metastases (n = 5) or the location of the metastasis to the portal vein (n = 2). The median (range) follow-up time from the date of diagnosis of the primary tumor was 40.1 (7.6-96.3) months. 21 patients (35%) died during follow-up: 17 patients due to disease progression, one patient because of complications during surgery and 3 patients because of other none disease-related causes. Median (SE) disease free survival of all the patients was 12.1 (1.9) months and the median (SE) overall survival was 23.5 (1.8) months. One year survival was 93% and 3-year survival 72%.

### RT-PCR

Bone marrow samples of 16 patients could not be analysed with RT-PCR because of the low amount of harvested mononuclear cells. RT-PCR positivity was found in 9 of 44 of the patients (20%). A positive RT-PCR test was seen in 6 of 32 patients (19%) who underwent a surgical resection compared to 3 of 12 inoperable patients (25%). Characteristics of the patients analyzed with RT-PCR and CK-ICC are shown in Table 1.

**Table 1:** Characteristics of the patients analyzed with RT-PCR and CK-ICC.

	RT-PCR		CK-ICC	
		
	positive N = 9	negative N = 35	positive N = 15	negative N = 31
Male	5 (56)	26 (74)	12 (80)	20 (65)
Age (years), mean (se)	63.4 (3.7)	60.9 (1.3)	62.9 (2.3)	60.2 (1.6)
TNM stage of primary tumor				
1	1 (11)	3 (9)	2 (13)	1 (3)
2	1 (11)	6 (17)	2 (13)	4 (13)
3	3 (33)	9 (26)	5 (33)	8 (26)
4	4 (45)	17 (49)	6 (40)	18 (58)
Time span between PT and LM (months)				
< 12	6 (67)	20 (57)	8 (53)	23 (74)
> 12	3 (33)	15 (43)	7 (47)	8 (26)
Preoperative systemic chemotherapy				
No	4 (44)	25 (71)	12 (80)	18 (58)
Yes	5 (56)	10 (29)	3 (20)	13 (42)
Serum CEA level				
< 200 ug/l	5 (55)	30 (86)	13 (87)	25 (81)
> 200 ug/l	1 (11)	3 (9)	1 (7)	2 (7)
not assessed	3 (33)	2 (5)	1 (7)	4 (14)
No. of liver metastases				
1	3 (33)	14 (40)	5 (33)	12 (39)
> 1	6 (67)	21 (60)	10 (67)	19 (61)
Diameter of liver metastases (cm)				
< 5	7 (77)	28 (80)	12 (80)	26 (84)
> 5	2 (33)	7 (20)	3 (20)	5 (16)
MSKCC clinical risk score				
0-2 (low)	4 (44)	24 (69)	12 (80)	20 (65)
≥ 3 (high)	5 (56)	11 (31)	3 (20)	11 (35)
Death	6 (67)	10 (29)	5 (33)	14 (45)

Data presented in number (%), unless otherwise stated.

*Abbreviations; PT, primary tumor; LM, liver metastases; CEA, carcino embryonic antigen; MSKCC, memorial sloan-kettering cancer center.

### CK-ICC

Due to a low number of harvested mononuclear cells, bone marrow samples of 14 colorectal liver metastases patients could not be analysed using ICC combined with automated microscopy to identify disseminated tumor cells. 15 of the 46 patients (33%) had a positive ICC test. ICC bone marrow analysis resulted in the presence of tumour cells in 13 of 37 patients (35%) who underwent a curative resection and 2 of 9 patients (22%) who finally underwent no surgical resection (only abdominal exploration).

### MSKCC-CRS

Primary tumor stage showed no correlation with the detection of tumor cells in bone marrow. High risk score-patients (3-5) according to the MSKCC-CRS showed disseminated tumor cells (RT-PCR) in 31% compared to 15% in the low risk group (0-2). CK-ICC positivity was found in 21% of the high-risk score-patients compared to 38% of the low risk score-patients.

### Pre-operative chemotherapy

Bone marrow of patients receiving neo-adjuvant chemotherapy was CK-ICC positive in 19% versus 40% in patients not receiving chemotherapy. RT-PCR positivity was found in 33% of the patients with chemotherapy compared to 14% of the patients without chemotherapy. Combining the two techniques (CK-ICC and/or RT-PCR positive), 57% of bone marrow was negative in patients who received chemotherapy and 54% in patients without pre-operative chemotherapy. Survival in the group receiving pre-operative chemotherapy was better than in the non chemotherapy group (p = 0.02). This was also seen when patients who did not undergo resection were excluded from this analysis (p = 0.03).

### Survival

**Figure 1:**
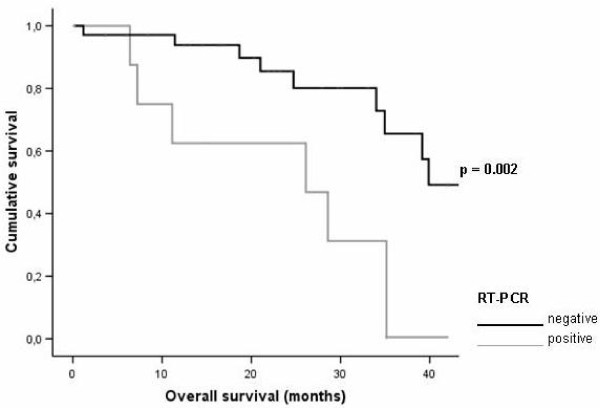
**Graphical representation of the relationship between reverse transcription-polymerase chain reaction (RT-PCR) status and overall survival in subjects after colorectal liver metastases surgery**.

**Figure 2:**
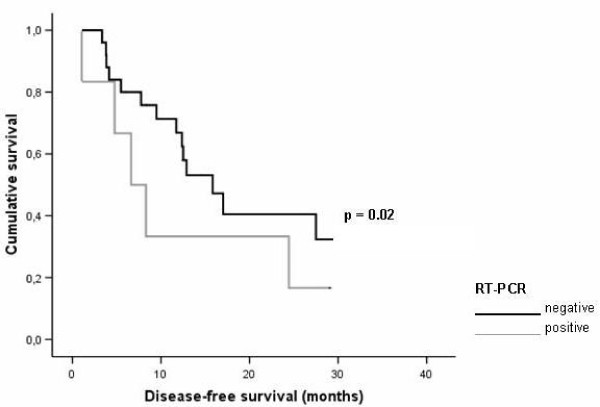
**Graphical representation of the relationship between reverse transcription-polymerase chain reaction (RT-PCR) status and disease free survival in subjects after colorectal liver metastases surgery**.

Patients with RT-PCR negative bone marrow had a significant better overall survival (Figure 1); HR 6.40, 95% CI 1.93-21.16, p = 0.002. This group also showed significant better disease-free survival after resection of their liver metastases (Figure 2); HR 4.11, 95% CI.1.33-12.58, p = 0.02.

CK-ICC positive bone marrow showed no significant difference in overall survival after resection (p = 0.24) neither a significant difference in disease-free survival (p = 0.86).

Combined RT-PCR and/or ICC positive analysis did not show any overall survival difference (p = 0.68) neither a difference in disease-free survival (p = 0.60). The low risk group according to the MSKCC-CRS (0-2) had a better overall survival (HR 3.32, 95%CI 1.14-9.67, p = 0.03) compared to the high MSKCC-CRS (3-5) patients. All HR were adjusted for age and sex.

When adjusted for MSKCC-CRS score, also a significant better overall survival was found in RT-PCR negative patients; HR 5.42, 95% CI.1.53-19.18, p = 0.009.

## Discussion

In our study disseminated tumor cells found in bone marrow by RT-PCR have prognostic value in patients scheduled for surgical resection of colorectal liver metastases. Patients with positive bone marrow by RT-PCR had an increased risk of cancer related mortality. Also adjusted for the MSKCC risk score, RT-PCR positivity showed a significant worse overall survival which reflects its additional medical value. In contrast, ICC did not predict outcome in these patients.

In our study the patients with positive RT-PCR bone marrow showed worse overall and disease-free survival after liver metastasis surgery. Similar results were found by *Koch et al *[15]. This study investigated bone marrow samples from 25 patients with colorectal liver metastases who underwent surgical resection and showed a positive RT-PCR test to be an independent prognostic factor for recurrence-free survival. The percentage RT-PCR positive bone marrow of the patients in our study (20.5%), is comparable with other studies, showing 16-27% RT-PCR positivity [15,18,19].

In contrast to RT-PCR, a positive ICC did not predict worse overall- and disease-free survival. Studies from *Bjornland et al *and *Schoppmeyer et al *had the same conclusion [14,17]. In contrast, DTCs detected with the CK-ICC in breast cancer patients are found to be of major prognostic significance [27]. It might be argued that the bone marrow in breast cancer patients not just reflects the metastatic load but is also a preferred site for metastases outgrowth. This may relate to the possibility that the bone marrow compartment offers a more fertile microenvironment for breast cancer cells than for colorectal cancer cells [28].

ICC positivity in bone marrow in our study is 32.6%. Differences in percentages of ICC positivity in colorectal cancer are reported in literature; *Schoppmeyer et al *found 55% CK positive cells in bone marrow [17], *Cohen et al *found 9.5-34% ICC positivity [29] and *Bjornland et al *found 8% ICC positivity [14].

The two methods used in our study to detect DTCs in liver metastatic colorectal cancer showed different results. A possible explanation is the hypothesis that only a small subset of tumor cells has the capacity to proliferate extensively and to outgrow to new tumors as increasing evidence supports [30,31]. Alternative detection methods, therefore may find disseminated cells that differ in their tumor initiating capacities. Another explanation may be that ICC detects only intact cells, while RT-PCR can also detect fragments of cells that are degraded in the circulation. Intact cells then may be considered biologically irrelevant (thus left alone by the immune system). Cells that would have been attacked by the immune system but escaped in distant organs may find a niche there to evolve into clinically manifest disease. Although highly speculative this same phenomenon has been described in minimal residual disease detection in lymph nodes [8], where RT-PCR is also more prognostic then ICC.

Earlier report from *Vlems et al *showed no positive bone marrow in 12 of the 20 patients who underwent chemotherapy and the authors therefore suggest chemotherapy prevents shedding or accelerates clearance of disseminated tumor cells [18]. However, we found no influence of preoperative chemotherapy on bone marrow positivity.

### Possible limitations and strength

The clinical relevance of the molecular detection of DTCs is possibly restricted by tumor cell heterogenity and differing sensitivity and specificity of each specific detection method. This may explain inconclusive findings from previous disseminated tumor cell studies. Despite the small number of patients, to the best of our knowledge our study is the largest study comparing both techniques, CK-ICC and RT-PCR, in patients with metastatic colorectal cancer.

## Conclusion

The presence of disseminated tumor cells in bone marrow of patients with colorectal cancer detected with RT-PCR does predict a worse clinical outcome in our study. Positive ICC did not find to have this predictive value. In the future, a more detailed and also functional analysis of the cells found in bone marrow of colorectal cancer patients may help in therapy selection and may give better prognostic information and as such contribute to patient management.

## Competing interests

The authors declare that they have no competing interests.

## Authors' contributions

FJV was responsible for sample collection, acquisition of patient data, statistical analysis, drafting and revising of the manuscript. WEM executed the ICC analysis in patient samples and was revising of the manuscript. AMR was responsible for sample collection, acquisition of patient data. GWP executed the RT-PCR analysis and was responsible for sample collection AML executed the ICC analysis in patient samples HJT was revising of the manuscript. RAT planned the study and revised the manuscript. GJL planned the study and revised the manuscript. All authors read and approved the final manuscript.

## Pre-publication history

The pre-publication history for this paper can be accessed here:

http://www.biomedcentral.com/1471-2407/10/153/prepub
